# Ligand-specific endocytic dwell times control functional selectivity of the cannabinoid receptor 1

**DOI:** 10.1038/ncomms5589

**Published:** 2014-08-01

**Authors:** Jacqueline Flores-Otero, Kwang H. Ahn, Francheska Delgado-Peraza, Ken Mackie, Debra A. Kendall, Guillermo A. Yudowski

**Affiliations:** 1 Department of Anatomy and Neurobiology, University of Puerto Rico, Medical Sciences Campus, PO Box 365067, San Juan 00936, Puerto Rico; 2 Department of Pharmaceutical Sciences, University of Connecticut, Storrs, Connecticut 06269, USA; 3 Institute of Neurobiology, University of Puerto Rico, Medical Sciences Campus, 201 Boulevard del Valle, San Juan 00901, Puerto Rico; 4 Department of Psychological and Brain Sciences, Gill Center for Biomedical Sciences, Indiana University, Bloomington, Indiana 47405, USA; 5 These authors contributed equally to this work

## Abstract

G protein-coupled receptors (GPCRs) are the major transducers of external stimuli and key therapeutic targets in many pathological conditions. When activated by different ligands, one receptor can elicit multiple signalling cascades that are mediated by G proteins or β-arrestin, a process defined as functional selectivity or ligand bias. However, the dynamic mechanisms underlying β-arrestin signalling remain unknown. Here by studying the cannabinoid receptor 1 (CB1R), we identify ligand-specific endocytic dwell times, that is, the time during which receptors are clustered into clathrin pits together with β-arrestins before endocytosis, as the mechanism controlling β-arrestin signalling. Agonists inducing short endocytic dwell times produce little or no β-arrestin signalling, whereas those eliciting prolonged dwell times induce robust signalling. Remarkably, extending CB1R dwell times by preventing endocytosis substantially increased β-arrestin signalling. These studies reveal how receptor activation translates into β-arrestin signalling and identify a mechanism to control this pathway.

## Abstract (web summary)

G-protein coupled receptors can signal through G-proteins or through β-arrestin, however mechanisms determining pathway selection remain unclear. Here the authors show that the duration of cannabinoid receptor clustering in clathrin coated pits prior to endocytosis determines the strength of β-arrestin signalling.

## Graphical abstract

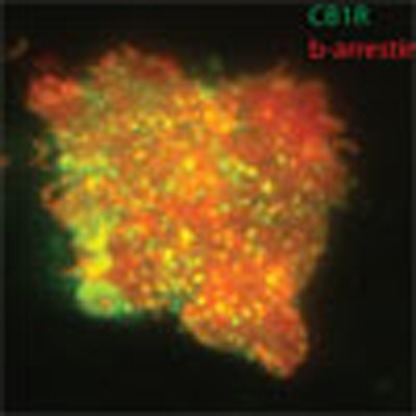

G protein-coupled receptors (GPCRs) are the largest family of signal transduction proteins in the mammalian genome and a major target of therapeutic drugs^1^,^2^. Their signalling includes both canonical pathways mediated by heterotrimeric G proteins, and non-canonical pathways mediated in part by β-arrestins^3^,^4^. β-arrestins are multifunctional scaffold proteins involved in desensitization, clathrin-mediated endocytosis (CME) and GPCR signalling^5^,^6^,^7^,^8^,^9^,^10^. Signalling mediated by β-arrestins is initiated with their recruitment to phosphorylated receptors at the plasma membrane and they can interact transiently or more permanently with GPCRs^11^,^12^,^13^,^14^,^15^.

Historically, GPCR signalling was considered linear and drug development programmes focused on the discovery of ligands that would either activate or block receptor function. However, this simplistic view has gradually evolved along with new pharmacological data. Today, we know that GPCR signalling can be pluridimensional. Different agonists acting on the same receptor can activate multiple and sometimes opposing signalling cascades, a process described as functional selectivity or ligand bias^4^,^10^,^12^. These agonists may stabilize different active conformations of the receptor that lead to selective coupling to signalling molecules. This pluridimensional efficacy holds tremendous therapeutic potential, and great effort is currently invested in the development of biased agonists that will selectively activate specific signalling cascades^6^,^16^,^17^,^18^. However, the dynamic molecular mechanisms underlying functional selectivity remain unknown, and painstaking pharmacological analysis is currently required to identify and develop more selective biased ligands, dramatically slowing the pace of drug discovery. To accelerate this process, a better understanding of the events that simultaneously control multiple signalling cascades from individual GPCRs is needed.

G-protein-signalling cascades have been exquisitely described from the molecular and mechanistic and, most recently, to the structural level^19^,^20^. By contrast, little is known about the mechanistic basis of β-arrestin-mediated signalling, although β-arrestin-biased ligands are more prevalent than originally thought and exhibit quite selective downstream outcomes. The current model of GPCR activation suggests that agonists induce unique receptor conformations and specific receptor phosphorylation patterns (bar codes) that are linked to β-arrestin recruitment and biased signalling^21^,^22^,^23^,^24^,^25^. How receptor activation translates into β-arrestin signalling and the dynamic events controlling β-arrestin signalling at the molecular level are not clearly known.

Here we combine quantitative live cell total internal reflection fluorescence (TIRF) microscopy and biochemical analysis to investigate endocytosis and biased signalling of the cannabinoid receptors 1 (CB1Rs) from endocytic pits. We demonstrate that agonists produce specific endocytic dwell times, that is, the time during which receptors are clustered with β-arrestins into endocytic pits before endocytosis, and dwell time controls β-arrestin-biased signalling. Prolonged endocytic dwell times produce sustained β-arrestin-biased signalling. Remarkably, extending dwell times by inhibiting endocytosis significantly increases β-arrestin signalling.

Ligand-specific dwell times have not been previously described and the physiological role of previously identified endocytic populations of clathrin-coated pits (CCPs) is currently unknown. These results identify a role for these multiple populations and describe a mechanism by which agonists control functional selectivity from the CB1R.

## Results

### Agonists induce specific CB1R endocytic dwell times

The CB1R is one of the most abundant receptors in the central nervous system and a key modulator of synaptic function^26^. CB1R agonists favour distinct receptor conformations that can lead to selective association with intracellular signalling pathways, a mechanism proposed for functional selectivity^27^,^28^,^29^. Following agonist-induced activation, β-arrestins are recruited to the plasma membrane to initiate CME^30^,^31^ and signalling. Given that CB1Rs and β-arrestins interact only transiently at the plasma membrane^31^,^32^, we hypothesized that specific agonist-induced receptor conformations control the duration of clustering of receptors and β-arrestins in endocytic pits as a mechanism to influence β-arrestin signalling.

**Figure 1:**
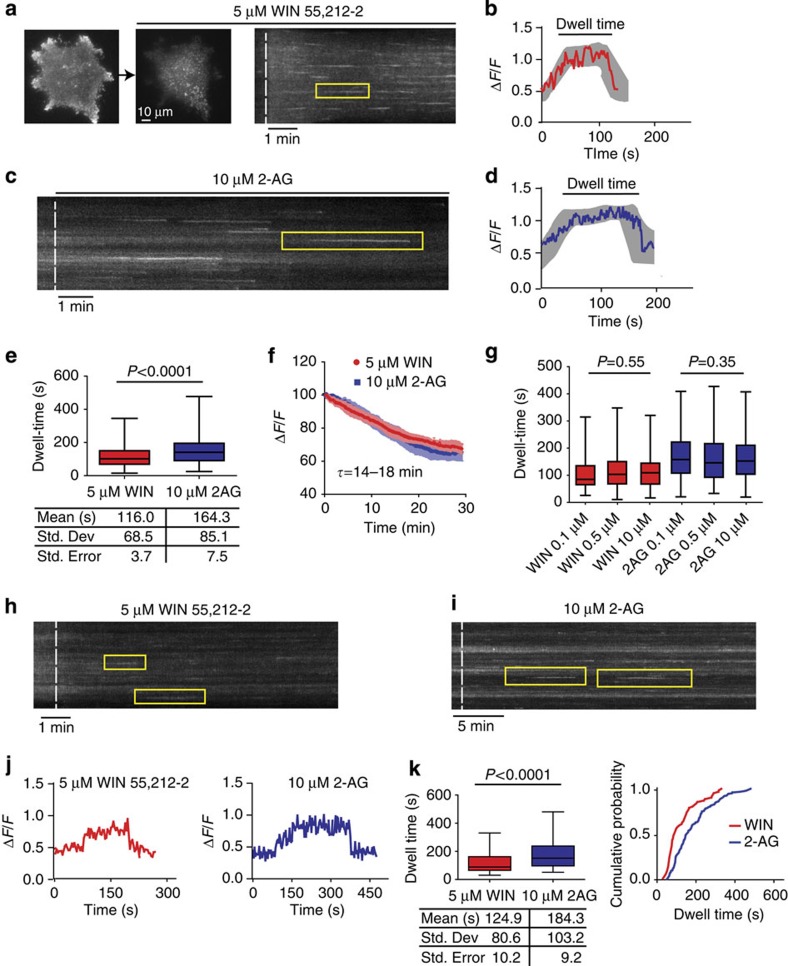
Agonists induce specific endocytic dwell times of the CB1R. (**a**) HEK293 cells expressing SEP-CB1Rs before and after 5μM WIN 55,212-2 imaged using TIRF. Kymographs from the cell surface show individual endocytic events (yellow rectangle). (**b**) Normalized fluorescence intensity from the event in **a**. s.d. from multiple normalized traces for 5μM WIN 55,212-2 is depicted in grey. (**c**) Representative kymograph showing individual SEP-CB1Rs endocytic events in the presence of 10μM 2-AG. (**d**) Normalized fluorescence intensity from an endocytic event elicited by 2-AG with s.d. from multiple events in grey. (**e**) Box and whiskers plot (median values with min/max range) showing dwell times of a single endocytic event elicited by 5μM WIN 55,212-2 and 10μM 2-AG (*n*=336 events per 12 cells and 244 events per 9 cells). (**f**) Endocytosis elicited by 5μM WIN 55,212-2 and 10-μM 2-AG was analysed by the decrease in total surface fluorescence intensity in HEK293 cells by time lapse TIRF microscopy (*n*=5 cells per treatment). (**g**) Dwell times were analysed for the indicated concentrations of agonist. No statistically significant difference was observed between concentrations (*n*=6–12 cells per condition). (The mean time±s.d. for 0.1 and 10 μM of WIN 55,212-2 was 106±63 and 117±88 s, respectively. The mean times for 0.1 and 0.5 μM of 2-AG was 177±87 and 159±80 s). (**h**) Kymograph from a hippocampal neuron expressing SEP-CB1Rs in the presence of 5 μM WIN 55,212-2. (**i**) Kymograph from a hippocampal neuron incubated with 10 μM 2-AG. (**j**) Representative traces of normalized fluorescence from individual endocytic events. (**k**) Box and whiskers plot (median values with min/max range) and cumulative probability graphs from SEP-CB1Rs dwell times elicited by the indicated agonists in hippocampal neuronal cultures (*n*=84 events per 7 cells and 109 events per 10 cells).

To test this hypothesis, we utilized TIRF microscopy of live cells to investigate the endocytosis of CB1Rs at the single pit level^33^. CB1Rs tagged at the extracellular amino terminus with the pH-sensitive super-ecliptic phluorin^34^,^35^ (SEP-CB1R) stably expressed in HEK293 cells were exposed to saturating concentrations of either the synthetic agonist WIN 55,212-2 or the endogenous eicosanoid 2-arachidonoylglycerol (2-AG), both considered to be high-efficacy agonists of G protein activation^36^,^37^. We found that 10μM 2-AG produced significantly longer endocytic dwell times, defined as the time receptors are clustered into endocytic pits before endocytosis, when compared with 5μM WIN 55,212-2 (Fig. 1a–e). Quenching experiments showed that all SEP-CB1R fluorescence was rapidly reduced by addition of MES, pH 5.5 to the imaging media. This indicated that the endocytic clusters analysed in our experiments are indeed at the cell surface and not dwelling in vesicles below the plasma membrane (Supplementary Fig. 1 and Supplementary Movie 1).

Distinct populations of CCPs have been reported at the plasma membrane: short-lived populations (*τ*<20–30 s) associated with abortive events and long-lived populations (*τ*>30–180 s) associated with productive endocytic events^38^,^39^. For GPCRs, their structure and ubiquitination levels have been shown to control the maturation process of endocytic pits^40^,^41^,^42^,^43^. However, it is not currently known whether agonists can control the kinetics of endocytic dwell times or whether the same receptor can adopt multiple dwell times.

Thus, next we sought to test whether the variability observed in dwell times reflects differences in the endocytic efficacy of the agonists or their binding properties to the receptors. First, we analysed the endocytosis of CB1Rs induced by 5μM WIN 55,212-2 and 10μM 2-AG, the same concentrations in the same cells where specific dwell times were observed. TIRF time-lapse microscopy was performed and surface fluorescence was analysed to determine the total rate of receptor removal from the cell surface (Fig. 1f). Results indicate that both agonists induced endocytosis of SEP-CB1R to the same extent with similar kinetics (*τ* =14–18 min; *P*=0.07 unpaired two-tailed *t*-tests throughout the manuscript unless stated). Second, we analysed whether dwell times were dependent on the concentration of the ligand. We analysed SEP-CB1R dwell times at concentrations below and close to the ligand Ki and also at near-saturating concentrations. The Ki values for WIN 55,212-2 and 2-AG were reported previously as 10–200 and 100–500 nM, respectively, with both agonists having similar potency and efficacy for GIRK current activation^44^,^45^,^46^,^47^,^48^. Analysis of multiple endocytic events across several experiments indicated that dwell times were not significantly different for the concentrations tested for each ligand (Fig. 1g). These results indicate that the time receptors are clustered into endocytic pits do not correlate with the endocytic efficacy or agonist concentration, suggesting a new role for dwell times during the endocytic process.

### Ligand-specific dwell times are a conserved mechanism

Previous studies have shown that CB1R function is dramatically sensitive to its cellular environment and results obtained in heterologous systems do not always replicate in more complex cells such as primary neuronal cultures^49^,^50^. To test whether ligand-specific dwell times are conserved in a more complex cellular environment, we transfected dissociated hippocampal neurons with SEP-CB1Rs at DIV 4–5 and performed TIRF microscopy at DIV >15. This lag between transfection and imaging significantly reduced the expression of SEP-CB1R towards physiological levels (Supplementary Fig. 2A,B) and resulted in cellular localization comparable to endogenous receptors (Supplementary Fig. 2C,D). As observed in HEK293 cells, 5μM WIN 55,212-2 and 10 μM 2-AG elicited dramatically different CB1R dwell times in hippocampal neuronal cultures (Fig. 1h–k). Dwell times in neurons were not significantly different from those observed in HEK293 cells for the same ligand (*P*=0.35 for 5 μM WIN and *P*=0.07 for 10 μM 2-AG in HEK versus neurons). These results indicate that agonists can elicit specific endocytic dwell times that are intrinsic to the ligand–receptor interaction and not to their cellular environment.

**Figure 2:**
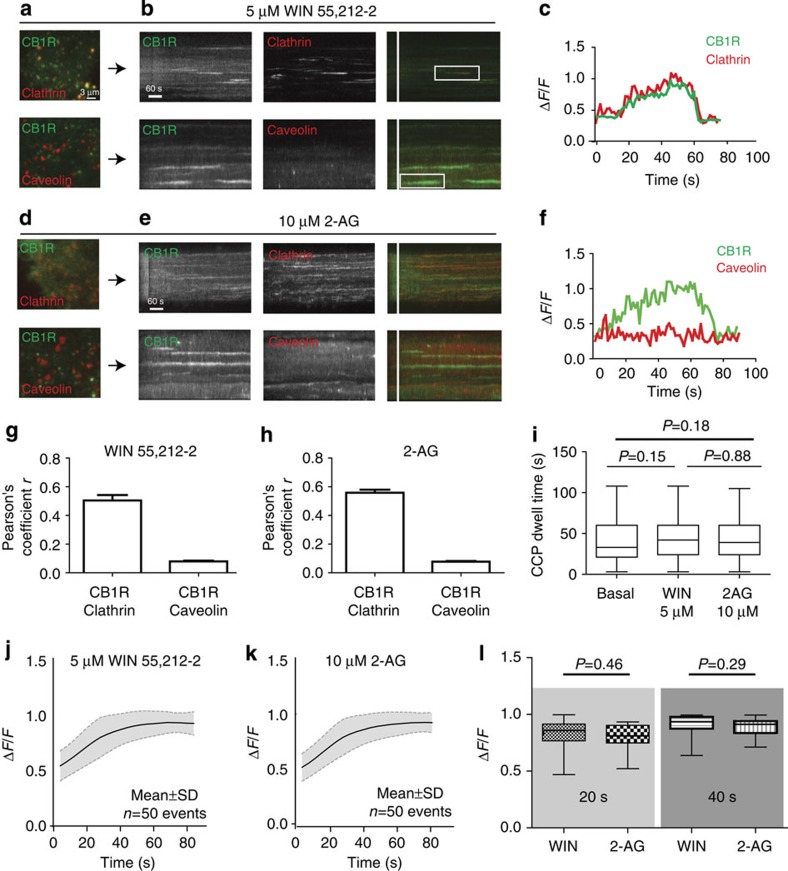
Agonists control the maturation process of CB1R clathrin-coated pits. (**a**) HEK293 cells co-expressing SEP-CB1Rs and DsRed-clathrin (top) or mCherry-caveolin-1 (bottom) incubated with 5μM WIN 55,212-2 and imaged under TIRF microscopy. Single frames show overlay after 5 min of incubation. (**b**) Kymographs showing individual SEP-CB1R endocytic event with clathrin (top, yellow traces) and caveolin-1 (bottom). (**c**) Representative normalized fluorescence intensity scan from the overlay of SEP-CB1Rs and DsRed-clathrin. (**d**) HEK293 cells expressing SEP-CB1Rs and either DsRed-clathrin (top) or mCherry-caveolin-1 (bottom) were incubated with 10μM 2-AG. Single frames after 5 min show the overlay. (**e**) Kymographs showing individual SEP-CB1R endocytic events with clathrin (top, yellow traces) and mCherry-caveolin-1 (bottom). (**f**) Representative fluorescence intensity trace from an individual SEP-CB1R endocytic event showing no colocalization with mCherry-caveolin-1. (**g**) Colocalization was analysed in multiple cells 5 min after treatments between CB1Rs and clathrin or CB1Rs and caveolin-1 (*n*=5–7 cells, error bars represent s.e.m. from multiple analyses). (**h**) Colocalization analysis after a 5-min treatment with 10μM 2-AG between SEP-CB1Rs and DsRed-clathrin or mCherry-caveolin-1 (*n*=6–9 cells, error represents s.e.m. from multiple analyses). (**i**) Clathrin-coated pit dwell times were analysed in HEK cells expressing only Ds-Red-clathrin under basal conditions or in the presence of the indicated ligands. (**j**) The mean intensity from the initial 20 s from events elicited with 5μM WIN 55,212-2, black shows the mean and grey s.d. (*n*=50 events). (**k**) The mean intensity from endocytic events elicited with 10μM 2-AG (*n*=70 events). (**l**) Box and whiskers analysis comparing normalized intensities from **j**,**k** at 20 s (left) and 40 s (right) after event onset (*P*=0.46 and *P*=0.29, respectively, plot indicates the median values and min/max range).

CME has been described as the major mechanism for ligand-induced endocytosis of the CB1R^31^,^51^,^52^. However, other studies suggested that CB1Rs expressed in heterologous cells can also colocalize with caveolin-1, and ligand-induced endocytosis can occur in parallel via CCPs and caveolar endocytosis^53^,^54^. As different dwell times could be associated with different endocytic pathways, we investigated which mechanism mediates ligand-specific dwell times of CB1Rs. We transiently transfected fluorescently tagged Ds-Red clathrin or mCherry-caveolin-1 into HEK293 cells stably expressing SEP-CB1Rs and analysed individual endocytic events elicited by 5μM WIN 55,212-2. Dual colour simultaneous TIRF imaging showed that SEP-CB1R endocytic pits colocalized only with clathrin, and not with caveolin-1 (Fig. 2a). Kymographs from endocytic movies and intensity measurements from individual events clearly show that SEP-CB1Rs associate exclusively with Ds-Red clathrin in the presence of WIN 55,212-2 (Fig. 2b,c). Next, we elicited endocytosis with 10μM 2-AG. Single frames from endocytic movies and their kymographs show that SEP-CB1R endocytosis is again associated with the recruitment of clathrin and not caveolin-1 (Fig. 2d,e). Fluorescence intensities from individual SEP-CB1R endocytic events failed to associate with mCherry-caveolin-1 (Fig. 2f). Colocalization analysis from individual endocytic events across multiple experiments indicated that CB1Rs were highly colocalized with clathrin (Pearson’s=0.5–0.55) when compared with caveolin (Pearson’s=0.07−0.05) for both agonists (Fig. 2g,h), strongly suggesting that CME is the endocytic mechanism underlying ligand-specific dwell times.

As modulation of dwell times could be mediated by the presence of CB1Rs or by a non-CB1R-mediated effect of the agonist, we analysed the dwell times of CCPs in HEK293 cells transfected with fluorescently tagged Ds-Red clathrin in the absence of SEP-CB1Rs. Dwell times were investigated in untreated cells or in the presence of 5μM WIN55,212-2 and 10μM 2-AG. Our results showed no significant difference in dwell times between untreated control and treated cells in the absence of SEP-CB1R (Fig. 2i). Importantly, dwell times obtained from CCPs closely resemble dwell times previously reported in the literature^55^,^56^,^57^.

CCPs traverse distinct steps during their lifetime before removal from the cell surface. Major events are assembly or pit initiation, maturation and final separation from the cell surface^55^. Growing evidence suggests that these steps can be regulated by lipid kinases and phosphatases that control the formation and maturation processes of CCPs^58^,^59^,^60^. To investigate which of these steps agonists modulate, we compared the changes in fluorescence intensity during the assembly and maturation processes of individual SEP-CB1R endocytic pits with similar kinetics. Fluorescence intensity traces from individual events were normalized to their maximum and compared for WIN 55,212-2 and 2-AG. Analysis of multiple events indicated that maximum intensities (maximum number of receptors) were reached with similar kinetics for both agonists, with no significant difference observed during the assembly phase (Fig. 2j–l, *τ* ~24 s). This suggests that the recruitment of receptors into individual endocytic pits occurs with similar kinetics; however, once the coated pits reach their maximum number of receptors (at maximum intensity), pits linger for variable periods of time depending on the ligand that activated the receptor. As we cannot identify changes at the single receptor level, we cannot rule out the possibility that individual receptors move in and out from single pits during dwell times as described for other cargoes^57^.

**Figure 3:**
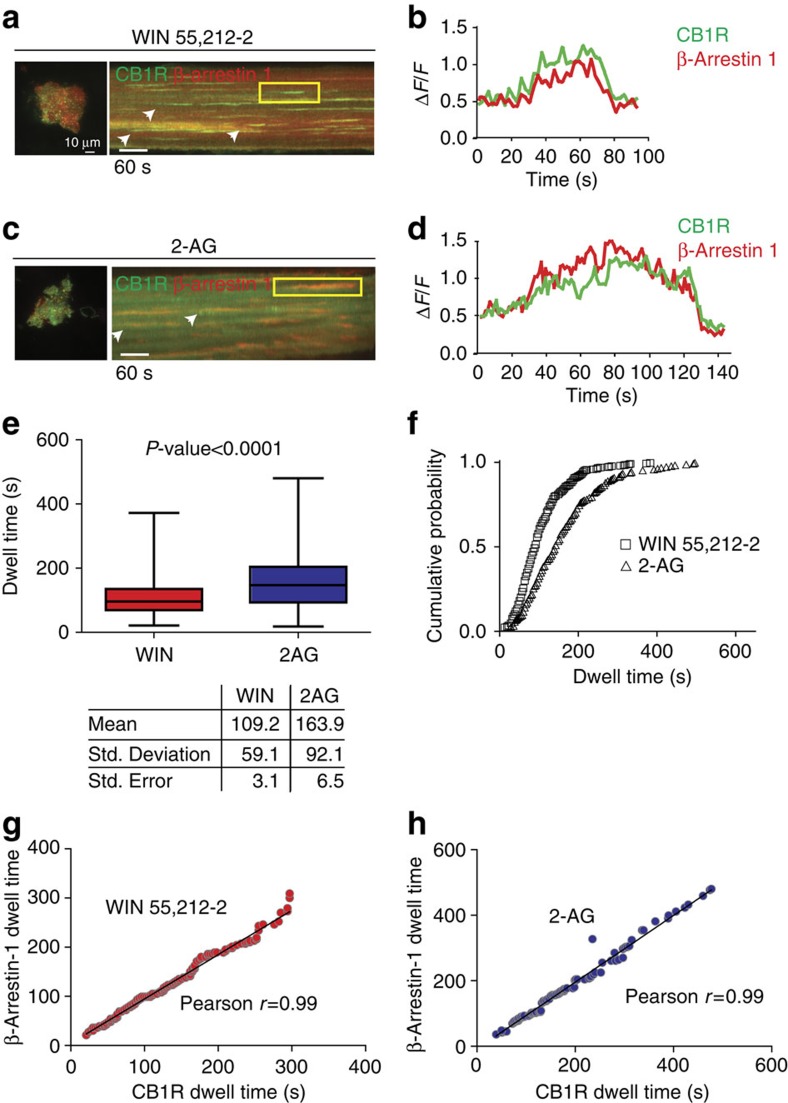
β-arrestin-1 is recruited and retained into CB1R CCPs with ligand-specific kinetics. (**a**) HEK293 cells co-expressing SEP-CB1R and mRFP-β-arrestin-1 were incubated with 5μM WIN 55,212-2 and analysed under dual-colour TIRF microscopy. Representative kymograph showing individual SEP-CB1R events colocalizing with mRFP-β-arrestin-1. (**b**) Individual normalized fluorescence intensity from SEP-CB1Rs and β-arrestin-1 during the endocytic event from **a** (yellow rectangle). (**c**) Representative kymograph from HEK293 cells expressing SEP-CB1R and mRFP-β-arrestin-1 in the presence of 10μM 2-AG. Individual SEP-CB1R endocytic events colocalize with β-arrestin-1. (**d**) Normalized fluorescence intensity from SEP-CB1Rs and mRFP-β-arrestin-1 from **c** (yellow rectangle). (**e**) Box and whiskers plot showing β-arrestin 1 dwell times into CCPs elicited by 5μM WIN 55,212-2 and 10μM 2-AG, *n*=342 events per 19 cells and 378 events per 21 cells (plot indicates the median values with min/max range). (**f**) Cumulative probability from β-arrestin 1 dwell times for 5μM WIN 55,212-2 and 10μM 2-AG. (**g**) Correlation between β-arrestin 1 dwell times and SEP-CB1R dwell times from the same endocytic event when elicited by 5μM WIN 55,212-2 (*P*<0.0001; *n*=305 endocytic events). (**h**) Correlation between β-arrestin 1 dwell times and SEP-CB1R dwell times from the same endocytic event when elicited by 5μM 2-AG (*P*<0.0001; *n*=197 endocytic events).

β-arrestins bind to activated GPCRs to initiate the endocytic process^11^,^12^,^13^,^14^. As agonists can determine the time receptors are clustered into endocytic pits with β-arrestins, we hypothesized that this regulation could mediate β-arrestin signalling. To test this hypothesis, first we investigated the recruitment kinetics of β-arrestin-1 into CB1Rs CCPs. We have previously shown that β-arrestin-1 mediates CB1R activation of ERK1/2 (ref. 61). Cells stably expressing SEP-CB1Rs were transfected with red fluorescent protein (RFP)-tagged β-arrestin-1 and incubated with either 5μM WIN 55,212-2 or 10μM 2-AG and imaged under TIRF microscopy. Upon ligand incubation, β-arrestin-1 was rapidly recruited to the plasma membrane and into individual endocytic pits as depicted in kymographs (Fig. 3a and Supplementary Movie 2). Normalized individual fluorescence traces indicated that β-arrestin-1 was recruited into SEP-CB1R endocytic pits and remained present throughout the endocytic process (Fig. 3b). As observed with SEP-CB1Rs, 2-AG elicited longer β-arrestin-1 dwell times when compared with WIN 55,212-2 (Fig. 3c,d). Analysis of multiple experiments from cells co-expressing RFP-β-arrestin-1 and SEP-CB1Rs showed a significant increase in the dwell time of β-arrestins in the presence of 2-AG (Fig. 3e,f). This increase in dwell time was comparable to those observed with SEP-CB1R alone, suggesting that the overexpression of RFP-β-arrestin did not have a deleterious effect on the endocytic process (Figs 1e and 3e). Correlation analysis between dwell times of CB1R and β-arrestin-1 from the same endocytic events indicated that both proteins are recruited and retained at CCPs with identical ligand-dependent kinetics (Fig. 3g,h).

**Figure 4:**
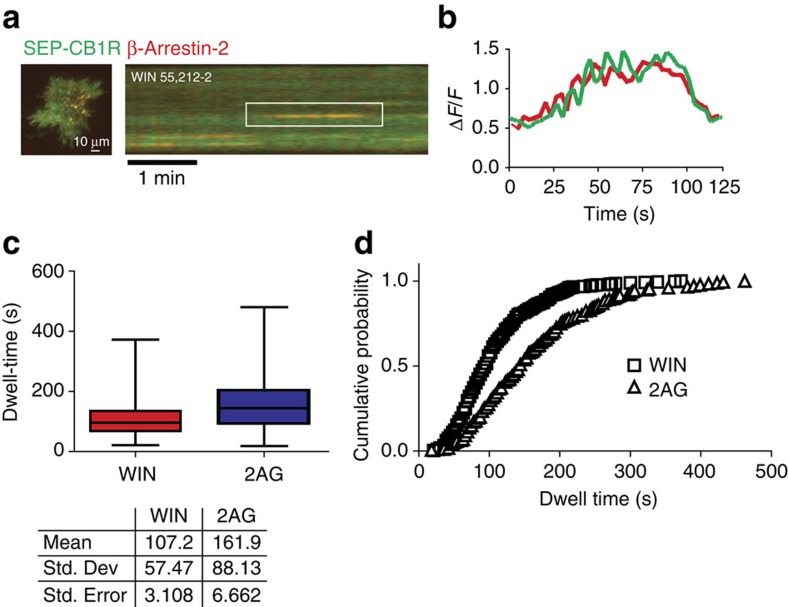
β-arrestin-2 is recruited and retained into CB1R CCPs with ligand-specific kinetics. (**a**) HEK293 cells co-expressing SEP-CB1R and mRFP-β-arrestin-2 were incubated with 5μM WIN 55,212-2 and analysed under dual-colour TIRF microscopy. Representative kymograph depicts an individual endocytic event and the colocalization between SEP-CB1R and mRFP-β-arrestin-2. (**b**) Individual normalized fluorescence intensity from SEP-CB1R and β-arrestin-2. (**c**) Box and whiskers plot (the median values and min/max range) showing β-arrestin-2 dwell times (*n*=304 events per 21 cells and 321 events per 15 cells). (**d**) Cumulative probability for β-arrestin 2 dwell times in the presence of 5μM WIN 55,212-2 and 10μM 2-AG.

β-arrestin-2 has previously been shown to be involved in CB1R internalization^28^. To investigate the recruitment kinetics of β-arrestin-2 to CB1R CCPs, HEK293 cells stably expressing SEP-CB1Rs were transfected with RFP-tagged β-arrestin-2. Recruitment was initiated by either 5μM WIN 55,212-2 or 10μM 2-AG. As previously observed with β-arrestin-1, β-arrestin-2 was efficiently recruited to the plasma membrane and into individual endocytic pits (Fig. 4a,b). Analysis of β-arrestin-2 dwell times at CCPs showed identical ligand-specific kinetics to those observed before (Figs 3e,f and 4c,d). These data show that both β-arrestins can be recruited and retained into CB1R-CCPs with ligand-dependent specificity.

### 2-AG signals through G proteins and β-arrestin-1

**Figure 5:**
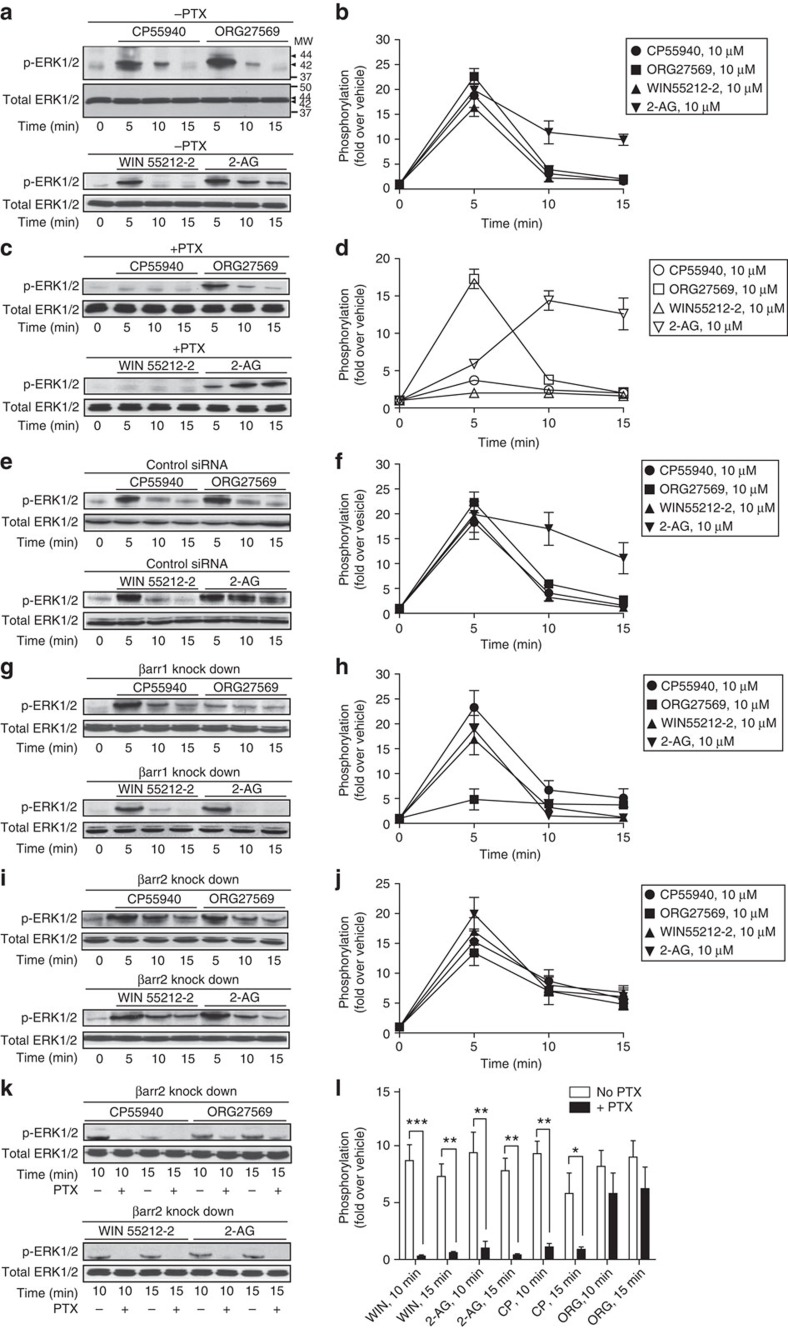
CB1R/β-arrestin-1 mediates the activation of ERK1/2 by 2-AG. (**a**) HEK293 cells expressing SEP-CB1Rs were exposed to 10μM CP55940, ORG27569, WIN 55212-2 and 2-AG for 5, 10 and 15 min. Cell lysates were analysed using western blots with phospho-ERK1/2 (p-ERK1/2) or total ERK1/2. (**b**) Quantified time course in **a** showing ERK1/2 phosphorylation levels induced by 10 μM of each compound. (**c**) HEK293 cells expressing SEP-CB1Rs were exposed to 10μM CP55940, ORG27569, WIN 55212-2 and 2-AG for 5, 10 and 15 min with PTX pre-treatment, respectively. (**d**) Quantified time course in **c** showing ERK1/2 phosphorylation levels from cells pretreated with PTX. (**e**,**g**,**i**,**k**) HEK293 cells co-expressing SEP-CB1R and either control (**e**), β-arrestin-1 (**g**), β-arrestin-2 (**i**) siRNAs or β-arrestin-2 siRNA with PTX pretreatment (**k**) were exposed to 10 μM of CP55940, ORG27569, WIN 55212-2 and 2-AG, respectively, as indicated. (**f**,**h**,**j**,**l**). Graphs in **f**,**h**,**j**,**l** provide the quantified ERK1/2 phosphorylation levels induced by 10 μM of each compound as shown in **e**,**g**,**i**,**k**. Data represent the mean±s.e.m. of at least three independent experiments.

To test the hypothesis that prolonged dwell times influence β-arrestin signalling, we investigated ERK1/2 phosphorylation in response to WIN 55,212-2 and 2-AG along with the agonists CP55940 and its ago-allosteric modulator ORG27569 for comparison. We have previously demonstrated that ERK1/2 can be activated via CB1Rs in both a G-protein-dependent and G-protein-independent manner^28^,^61^,^62^. All four compounds induced ERK1/2 phosphorylation that peaked at 5 min (Fig. 5a,b). Most strikingly, 2-AG treatment resulted in sustained phosphorylation that was still substantial at 15 min (Fig. 5a bottom and Fig. 5b), while the phosphorylation elicited by CP55940, ORG27569 and WIN 55,212-2 drastically decreases after 5 min.

To establish specificity, HEK293 cells lacking CB1Rs did not show changes in ERK1/2 phosphorylation upon treatment of each of the four compounds, indicating that the phosphorylation pattern shown in Fig. 5a is CB1-mediated (Supplementary Fig. 3D). We also analysed whether ORG27569 can induce endocytic events. Interestingly, 10μM ORG27569 elicited a very small number of endocytic events reflecting its ago-allosteric function. Remarkably, the dwell times elicited by ORG27569 were statistically indistinguishable from those observed with 2-AG (Supplementary Fig. 3C).

To evaluate the involvement of Gi/o proteins in ERK1/2 phosphorylation, we treated cells expressing SEP-CB1Rs with pertussis toxin (PTX). Consistent with previous findings^56^, PTX attenuated CP55940-induced ERK1/2 phosphorylation, while it showed no effect on ORG27569-induced phosphorylation (Fig. 5c top). PTX treatment also abolished WIN 55,212-2-induced ERK1/2 phosphorylation. Strikingly, PTX treatment inhibited 2-AG-induced ERK1/2 phosphorylation at the early time point (5 min), but not the later time points (10 and 15 min; Fig. 5c bottom and 5d). This suggests that 2-AG induces ERK1/2 activation via multiple pathways and only activation at the early time point is mediated by Gi proteins.

To test whether β-arrestins mediate 2-AG-induced ERK1/2 phosphorylation at later time points, we used silencing technology to reduce the expression of endogenous β-arrestin-1 and β-arrestin-2. Co-transfection with SEP-CB1Rs and negative control short interfering RNA (siRNA) exhibited a similar pattern of ERK1/2 phosphorylation (Fig. 5e,f) to controls (Fig. 5b). Consistent with previous data^28^, Fig. 5g shows that while the reduced expression of β-arrestin-1 exhibited little effect on CP55940-induced ERK1/2 phosphorylation, it substantially abrogated ORG27569-induced ERK1/2 phosphorylation, indicating that this phosphorylation is mediated by β-arrestin-1. WIN 55,212-2-induced ERK1/2 phosphorylation remained unaffected with the reduced expression of β-arrestin-1. Most strikingly, silencing β-arrestin-1 resulted in a significant decrease in the level of phosphorylation induced by 2-AG at the later time points (10 and 15 min) without affecting its phosphorylation at the 5-min time point (Fig. 5g,h). The effectiveness of isoform-specific silencing of β-arrestin 1 and 2 was confirmed (Supplementary Fig. 3E). Taken together, these results show that 2-AG induces a maximal Gi/o-mediated ERK1/2 phosphorylation at 5 min followed by a sustained ERK1/2 activation at the later time points (10 and 15 min) mediated by β-arrestin-1 providing the first reported case of canonical and non-canonical signalling pathways induced by the endogenous agonist 2-AG via CB1R.

Reduction of β-arrestin-2 using siRNA did not have a substantial effect on CP55940-, ORG27569- and WIN 55,212-2-induced ERK1/2 phosphorylation when compared with controls (Fig. 5i), although these agonists resulted in a small but sustained level of ERK1/2 phosphorylation at the later time points (10 and 15 min) compared with those shown by control siRNA transfection (Fig. 5j). This may reflect a need for β-arrestin-2 for internalization^31^,^32^ and reduced internalization may allow for extended Gi/o signalling. To test this possibility, we treated cells expressing SEP-CB1Rs with PTX. PTX treatment inhibited CP55940- and WIN 55,212-2-induced extended ERK phosphorylation suggesting that the extended ERK1/2 phosphorylation is Gi-mediated (Fig. 5k,l). In contrast, the substantially sustained levels of ERK1/2 phosphorylation at the later time points induced by 2-AG treatment (Fig. 5e bottom) was diminished to a level similar to that of CP55940, ORG27569 or WIN 55,212-2 treatment. It is possible that the reduced expression of β-arrestin-2 results in inhibition of CCP formation, thus abrogating the β-arrestin signalling that occurs in endocytic pits but prolonging ongoing Gi/o signalling. Interestingly, PTX treatment further inhibited 2-AG-induced ERK phosphorylation (Fig. 5k) suggesting that the diminished level of phosphorylation resulting from β-arrestin-2 knockdown is Gi-mediated.

### Extension of dwell times enhances β-arrestin signalling

**Figure 6:**
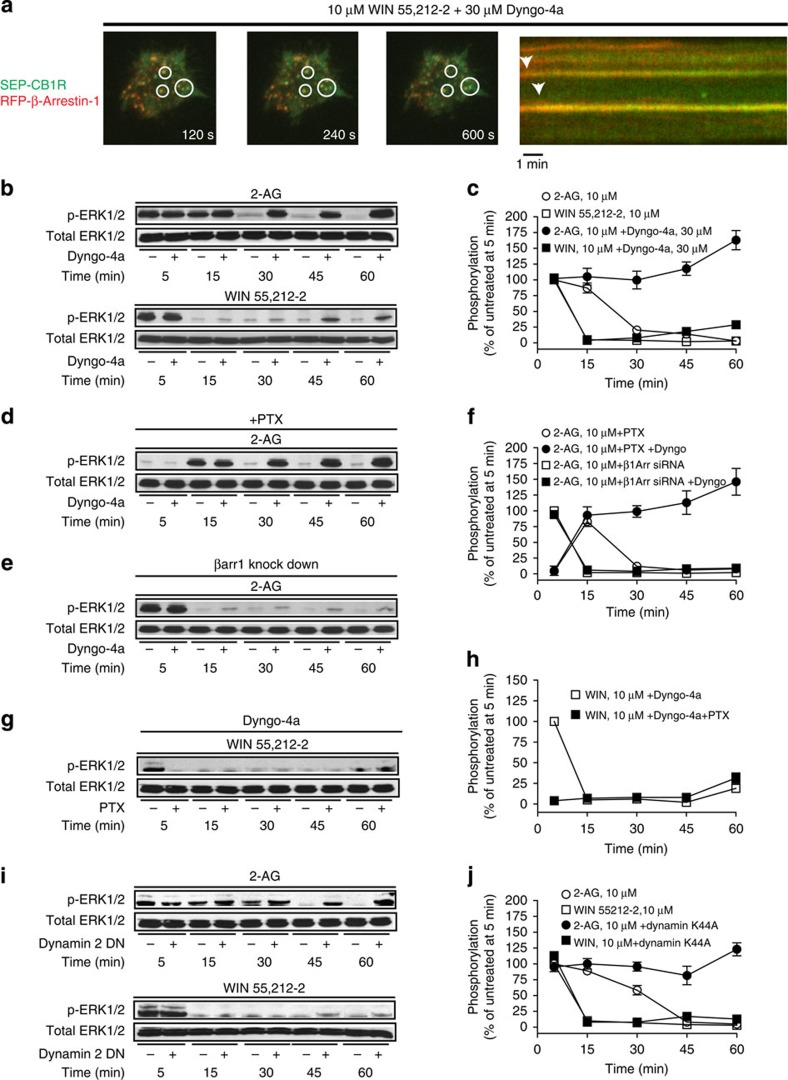
CB1R dwell times control β-arrestin signalling. (**a**) HEK293 cells expressing SEP-CB1R were preincubated with 30μM dyngo-4a and imaged under TIRF before and after bath application of 10μM WIN55212-2. Representative kymograph shows prolonged SEP-CB1R dwell times in the presence of dyngo-4a. (**b**) HEK293 cells expressing SEP-CB1R were pre-incubated with 30μM dyngo-4a before exposure to agonists and compared with no dyngo-4a treatment. (**c**) Graph provides the quantified ERK1/2 phosphorylation induced by 10 μM of each compound shown in **b**. Data are expressed as a percentage of the level of phosphorylation at 5 min for each compound without dyngo-4a pre-treatment. (**d**) HEK293 cells expressing SEP-CB1Rs with and without dyngo-4a pretreatment were exposed to 10μM 2-AG as indicated with PTX pre-treatment. (**e**) HEK293 cells co-expressing SEP-CB1R and β-arrestin-1 siRNAs with and without dyngo-4a pretreatment were exposed to 10μM 2-AG as indicated. (**f**) Quantified time course in **d**,**e** showing ERK1/2 phosphorylation levels induced by 10μM 2-AG for 5, 15, 30, 45 and 60 min for cells either pre-treated with PTX or co-transfected with β-arrestin-1 siRNA. (**g**) HEK293 cells expressing SEP-CB1R were treated with PTX (or no PTX as a control), and then incubated with 30μM dyngo-4a before subsequent exposure to WIN 55,212-2. (**h**) Graph provides the quantified ERK1/2 phosphorylation induced by 10μM WIN 55,212-2 in the absence of PTX treatment shown in **g**. (**i**) HEK293 cells expressing SEP-CB1R with or without dominant-negative dynamin 2 K44A were exposed to agonists. (**j**) Graph provides the quantified ERK1/2 phosphorylation induced by 10 μM of each compound shown in **i**. All data are expressed as a percentage of the level of phosphorylation at 5 min for 2-AG without dyngo-4a pre-treatment and represent the mean±s.e.m. of at least three independent experiments.

To directly test the hypothesis that dwell times dictate β-arrestin-mediated ERK1/2 signalling, we pre-treated cells with the dynamin inhibitor dyngo-4a. Inhibition of endocytosis dramatically prolonged SEP-CB1R dwell times throughout the imaging sessions (Fig. 6a) and, according to our model, it should enhance β-arrestin-mediated signalling. Since 2-AG exhibited a significantly longer endocytic dwell time than WIN 55,212-2 (Fig. 1e) and sustained β-arrestin-mediated ERK1/2 signalling (Fig. 5), the effect of prolonging the endocytic dwell time on 2-AG-induced ERK1/2 phosphorylation was evaluated up to 60 min. Figure 6b shows that 2-AG treatment (no dyngo-4a) exhibited a sustained phosphorylation pattern of ERK1/2 up to 15 min, while the phosphorylation by WIN 55,212-2 drastically decreases after the 5-min peaks. Strikingly, pre-treatment with dyngo-4a resulted not only in an extension but also in an increase in 2-AG-induced ERK1/2 phosphorylation (Fig. 6b,c). WIN 55,212-2 treatment showed essentially no increase in ERK1/2 phosphorylation in the presence of dyngo-4a, except for a small increase at later time points (45 and 60 min). One possible explanation is that prolonging dwell times results in increased interactions with either G protein or β-arrestin for ERK1/2 signaling.

Finally, to determine whether the increase in ERK1/2 phosphorylation by 2-AG in the presence of dyngo-4a was mediated by β-arrestins, cells were incubated with PTX or co-transfected with β-arrestin-1 siRNA. Figure 6d shows that preincubation with PTX inhibited 2-AG-induced ERK1/2 phosphorylation at 5 min but had no effect during the later time points between 15 and 60 min. In contrast, silencing of β-arrestin-1 expression resulted in a substantial decrease in the level of phosphorylation induced by 2-AG at the later time points between 15 and 60 min without affecting its phosphorylation at the 5-min time point (Fig. 6e,f). To investigate whether prolonging endocytic dwell times with dyngo-4a treatment results in an increase in ERK1/2 phosphorylation in the absence of Gi protein, we treated PTX-pretreated HEK293 cells expressing SEP-CB1R with dyngo-4a. WIN 55,212-2 treatment failed to show any increase in ERK1/2 phosphorylation in the presence of dyngo-4a and PTX compared with those without PTX treatment (Fig. 6g,h). These data suggest that WIN 55,212-2 promotes the CB1 conformation that couples to Gi but not to β-arrestin

To further confirm the correlation between longer endocytic dwell times and extended β-arrestin signalling induced by dyngo-4a, we expressed a dynamin 2-dominant-negative mutant. Expression of the dominant-negative dynamin also substantially extended ERK1/2 phosphorylation, similarly to dyngo-4a treatment, upon 2-AG treatment (Fig. 6i top and Fig. 6j). In contrast, WIN 55,212-2 treatment showed essentially no increase in ERK1/2 phosphorylation upon co-expression of the dynamin 2-dominant-negative mutant (Fig. 6i bottom), also comparable to dyngo-4a treatment. Taken together, these results show that prolonging CB1R dwell times by the dynamin inhibitor dyngo-4a or expression of the dominant-negative dynamin 2 significantly increased 2-AG-induced activation of ERK1/2 mediated by β-arrestin-1.

## Discussion

Our results provide insights into the molecular events underlying functional selectivity during β-arrestin signalling from the CB1R. We demonstrate that agonists modulate how long receptors interact with β-arrestins in endocytic pits at the cell surface to control the extent of β-arrestin signalling. The synthetic agonist WIN 55,212-2 elicits short endocytic dwell times and activates mainly G proteins (G protein bias), while the endogenous agonist 2-AG elicits prolonged dwell times and promotes short-term G protein and longer-term β-arrestin signalling (as expected for a more balanced ligand). Ligand specificity of dwell time was identical in hippocampal neurons, while siRNA silencing technology and pertussis toxin treatment confirmed that activation of ERK1/2 was indeed selectively mediated by β-arrestin-1. β-arrestin-2 was also recruited to CCPs with similar kinetics, likely mediating receptor internalization based on earlier work. To further challenge our model, chemical and genetic extension of receptor dwell times resulted in a significant increase in β-arrestin-1-mediated signalling.

It is interesting that, although WIN 55,212-2 induces CB1R endocytosis via β-arrestin^32^, it failed to show β-arrestin-mediated signalling. Our results using PTX treatment and β-arrestin siRNA indicate that WIN 55,212-2-induced ERK1/2 phosphorylation is G-protein-mediated. In previous studies, we demonstrated that, although β-arrestin-biased ORG27569 and G protein-biased CP55940 (ref. 28) and WIN 55,212-2 (ref. 32) induced β-arrestin 2-mediated endocytosis, only ORG27569, but not CP55940-induced c-Src activation in a β-arrestin-dependent manner. Interestingly, Gyombolai *et al.*^32^ also showed no significant interaction of CB1R with β-arrestin-1 upon WIN 55,212-2 treatment, which is the key isoform for signalling for CB1R^28^. Collectively, while we cannot absolutely rule out the possibility that WIN 55,212-2 treatment induces other CB1R signalling cascades via β-arrestin, these data suggest that WIN55212-2 is a G protein-biased agonist and requires β-arrestin-2 for endocytosis only.

How are ligand-specific dwell times generated and how do they control signalling? As the CB1R is a member of the class A family and the interaction with β-arrestins occurs transiently at the plasma membrane before endocytosis, modulation of β-arrestin signalling would occur during the formation of CCPs and before their removal from the cell surface^31^,^32^. We hypothesize that endocytic agonists induce specific phosphorylation patterns or bar codes in the intracellular domain of the receptor, stabilizing specific conformations that promote differential interactions with β-arrestins at the plasma membrane while initiating signalling^23^,^24^,^63^. In this case, 2-AG would generate a receptor conformation and consequent phosphorylation bar code that will translate into a specific and prolonged interaction between receptor and β-arrestin-1. How can β-arrestins activate specific signalling cascades during the endocytic process? This requires a dynamic mechanism that could link distinct signalling molecules to correct receptor/arrestin combinations. The extended contact during prolonged dwell times could provide the necessary time for all the signalling components to be effectively recruited to β-arrestin-1 and activated. Although both WIN 55,212-2 and 2-AG induced the receptor endocytosis with a similar kinetics, 2-AG treatment resulted in prolonged β-arrestin-mediated signalling and endocytic dwell times. It will be interesting to investigate the kinetics of interaction between CB1R and each β-arrestin isoform^15^ and determine whether subsequent endosome signalling also occurs^64^,^65^. This study identifies the mechanisms by which CB1Rs controls β-arrestin signalling, and suggests modulation of receptor trafficking as a novel therapeutic strategy to control GPCR function.

## Methods

### Cell cultures and transfection

Human embryonic kidney cells (HEK293 cells) were obtained from ATCC (Manassas, VA, USA). They were maintained in complete growth Dulbecco’s modified Eagle’s medium (DMEM), supplemented with 10% fetal bovine serum (FBS), 0.1% penicillin–streptomycin and 2 mM glutamine (all from Life Technologies, Grand Island, NY, USA). Embryonic day 14 rat hippocampal tissue was obtained from BrainBits LLC (Springfield, IL, USA). Twenty-four hours post arrival, hippocampal tissue was dissociated in 1 × trypsin (Life Technologies) for 15 min at 37 °C before 4 ml of supplemented DMEM was added for 5 min at room temperature. Hippocampal neurons were triturated using a glass pipette. Cultures of 250,000 cells per 35-mm petri dish were plated and incubated 5 days before transfection. Before seeding, glass coverslips (30 mm) were coated with poly-D-Lysine and after detection of neuronal attachment, culture media was replaced for Neurobasal media (Life technologies). HEK293 cell and hippocampal neurons were transfected with 3–4 μg of SEP-CB1R cDNA (gift from Andrew Irving, University of Dundee and characterized here^34^ or co-transfected with 2–3 μg of SEP-CB1R: Ds-Red-Clathrin (CCP) light chain cDNAs; SEPCB1: mCherry-Caveolae cDNAs (gift from Mark von Zastrow, University of California, San Francisco) or SEP-CB1: RFP-β-Arrestin2 cDNAs (kindly provided by Dr. Ken Mackie). HEK293 cells were transfected using Effectene following the manufacturer’s protocol (Qiagen, Valencia, CA, USA), after DIV2-3 post culture. Hippocampal neurons were transfected using Lipofectamine 2000 (Life Technologies) according to the manufacturer’s protocol after DIV5 post culture. CB1R ligands such as WIN 55212-2 and 2-AG, 2-AG were purchased from Tocris (Tocris Bioscience, Bristol, UK).

### β-arrestin siRNA and dominant-negative dynamin experiments

HEK293 cells (ATCC) were maintained in DMEM supplemented with 10% FBS and 3.5 mg ml^−l^ glucose at 37 °C in 5% CO_2_. Transfection was carried out using lipofectamine (Life Technologies) according to the manufacturer’s instructions. Twenty-four hours post transfection, cells were washed and incubated for an additional 18 h in serum-free growth media. siRNA (Qiagen) transfection was carried out as previously described^28^. Briefly, HEK293 cells that were 40–50% confluent in a six-well plate were transfected with the plasmid encoding rat CB1 and 2.6 mg of siRNA (Qiagen) sequences targeting β-arrestin 1, β-arrestin 2 or non-silencing RNA duplex for control. Silencing of β-arrestin 1 and β-arrestin 2 expression was assessed by immunoblotting using anti-β-arrestin 1 (1:2,000; EMD Millipore, Billerica, MA, USA) and anti-β-arrestin 2 (1:1,000; Novus Biologicals, Littleton, CO, USA) antibodies, respectively. For the dominant-negative dynamin 2 experiments, the dynamin 2 K44A mutant was generated by site-directed mutagenesis (Stratagene, La Jolla, CA, USA) using the human dynamin 2 cloned into the pmCherryN1 vector (Addgene, Cambridge, MA, USA). HEK293 cells were co-transfected with SEP-CB1R and K44A dynamin 1-mCherry cDNAs using lipofectamine described above.

### Immunoblotting studies

Cells expressing HA-tagged rat CB1 receptor were washed twice and exposed to either 0.5 or 10 μM of the CB1 agonists CP55940, WIN 55212-2 or 2-AG, or the allosteric modulator ORG27569 for 5, 10 and 15 min. To observe the effect of PTX on ERK1/2 phosphorylation, cells were pretreated with 10 ng ml^−l^ for 16 h at 37 °C. In Dyngo-4a experiments to inhibit dynamin and endocytosis, cells were incubated with 30 μM Dyngo-4a for 15 min before agonist treatment. Cells were exposed to 10μM 2-AG or WIN55212-2 for 5, 15, 30, 45 and 60 min, washed with ice-cold phosphate-buffered saline (PBS) and lysed in ice-cold lysis buffer consisting of 150 mM NaCl, 1.0% IGEPAL CA-630, 0.5% sodium deoxycholate, 0.1% SDS, 50 mM Tris, pH 7.5 and a protease inhibitor cocktail 4-(2-aminoethyl)benzenesulfonyl fluoride, pepstatin A, E-64, bestatin, leupeptin and aprotinin; Sigma, St Louis, MO, USA). Solubilized cell extracts were centrifuged at 18,500 *g* for 15 min at 4 °C. The supernatant was transferred to a fresh tube and heated at 95 °C for 3 min. In all, 15 μg of total proteins were resolved with SDS–PAGE gel electrophoresis in 10% gels and transferred onto polyvinylidene fluoride membrane. After incubating with blocking reagent (Fisher Scientific, Pittsburgh, PA, USA), the membrane was incubated for 1 h at room temperature with the primary antibody (1:3,000 phospho-p44/42 and p44/42 antibodies; Cell Signaling Technology, Danvers, MA, USA). After extensive washing with PBS, the membrane was incubated with anti-rabbit peroxidase-conjugated secondary antibody (1:5,000; Cell Signaling Technology) for 45 min at room temperature. The specific immunoreactivity was visualized using the SuperSignal West Femto Chemiluminescent Substrate System (Thermo Fisher Scientific, Rockford, IL, USA) according to the manufacturer’s instructions. Immunoreactive bands of phospho-ERK1/2 were quantified by densitometric analysis using the ImageJ programme ( http://rsb.info.nih.gov/ij/) and normalized to the intensity of total-ERK1/2. Data are expressed as a fold increase above the basal level of phosphorylation. Uncropped blots of the most important western blots are included in the Supplementary Fig. 4.

### TIRF microscopy

TIRFM was performed in HEK293 cells and hippocampal neurons on DIV5 and DIV12, respectively, utilizing a Motorized Nikon Ti-E inverted microscope with a CFI-Apo × 100 1.49 oil TIRF objective lens with colour correction and a motorized stage with perfect focus(Melville, NY, USA). Light sources were 488- and 561-nm Coherent sapphire lasers (Coherent Inc., Santa Clara, CA, USA) with 50 and 100 mW, respectively. The microscope was coupled to an iXonEM+DU897 back illuminated EMCCD camera (Andor, Belfast, UK). Imaging settings were kept constant throughout our imaging: readout speed: 10 Hz, exposure time: 100 ms every 3 s, EM gain 300, binning: 1 × 1, image: 512 × 512, BitDepth=14 bits, temperature: −75 and laser power: 10%. Cells were kept at 37 °C with a Stable Z stage and objective warmer (Bioptechs, Butler, PA, USA).

Cells were gently rinsed three times with OptiMem supplemented with 20 mM HEPES (Life Technologies) and kept in the incubator for 10–30 min to acclimate before imaging. TIRFM recording was conducted in the same imaging media for 1–3 min under basal condition (without any treatment) and was followed by bath application of selected ligand using a custom-built perfusion chamber. Total time of live-imaging visualization and recording was less than 30 min.

### Image processing and analysis

Analysis of SEP-CB1, Ds-Red-CCP, mCherry-Caveolae or RFP-β-Arrestin2 endocytic events was performed as described before^33^,^66^,^67^. Briefly, raw images were first background-subtracted and flat field-corrected^33^. Individual endocytic events were quantified by an observer blinded to experimental details, multiple times manually and using the particle tracking algorithm two-dimensional spot tracker^68^. Individual event location, time and fluorescence profile were logged and recorded. Individual endocytic events were identified and scored according to the following criteria: (1) individual events appeared and disappeared within the time series; (2) endocytic events displayed limited movement in the *x* and *y* axes as described for clathrin endocytic pits during their maturation phase^38^,^55^,^69^; and (3) the events did not collide or merge with other structures. Dwell times were calculated as the time between the first frame where spot tracker detected an event above background fluorescence levels and the last. As the fluorescence from individual events can fluctuate and the algorithm from the tracking software can misinterpret endocytic events, we manually verify all individual events after automated analysis. Figures of endocytic events are shown through orthogonal views of image series as kymographs. In the kymographs, horizontal lines illustrate the receptor’s endocytic event. The beginning of the horizontal line represents the time at which the receptor is at the surface of the membrane, the end of the horizontal line represents the time at which the receptor is internalized and the length of the horizontal line refers to the duration of the receptor at the cell surface before endocytosis occurs (endocytic lifetime or dwell time). Endocytic time constants from bulk endocytosis (Fig. 1f) where determined by fitting raw fluorescence data to a single exponential decay function± s.d. All other data are expressed as means±s.e.m unless stated. Unpaired two-tailed Student’s *t*-tests were used to test for statistical significance. Statistical analyses including Pearson’s correlation between dwell times were calculated utilizing the GraphPad Prism Software (La Jolla, CA, USA). Box and whiskers plot represent minimum and maximum values, the box extends from 25 to 75% with the mean value.

## Additional information

**How to cite this article:** Flores-Otero, J. *et al.* Ligand-specific endocytic dwell times control functional selectivity of the cannabinoid receptor 1. *Nat. Commun.* 5:4589 doi: 10.1038/ncomms5589 (2014).

## Supplementary Material

Supplementary Figures Supplementary Figures 1-4

Supplementary Movie 1 Surface SEP-CB1Rs quenching. Simultaneous dual fluorescence TIRF microscopy series (10Hz) from a HEK cell co-expressing SEP-CB1R and RFP-β-arrestin-2 were incubated with 5 μM WIN 55,212-2 to induce receptor clustering and endocytosis. Perfusion of MES pH5.5 resulted in a rapid decrease in SEP-CB1R fluorescence.

Supplementary Movie 2 Imaging CB1Rs endocytosis by TIRF microscopy. Simultaneous dual fluorescence TIRF microscopy series (0.3Hz) from a HEK cell co-expressing SEP-CB1R and RFP-β-arrestin-2. Endocytosis was initiated with 5 μM WIN 55,212-2, resulting in receptor clustering, beta-arrestin recruitment and endocytosis. Total running time= 30 minutes.
