# Supplementary material for: Ligand-specific endocytic dwell times control functional selectivity of the cannabinoid receptor 1
Source: Nat Commun. 2014 Aug 1;5:4589. doi: 10.1038/ncomms5589 (PMC4227836; doi:10.1038/ncomms5589)
Supplement: Supplementary Figures — 1-4 [file ncomms5589-s1.pdf]

Supporting Information

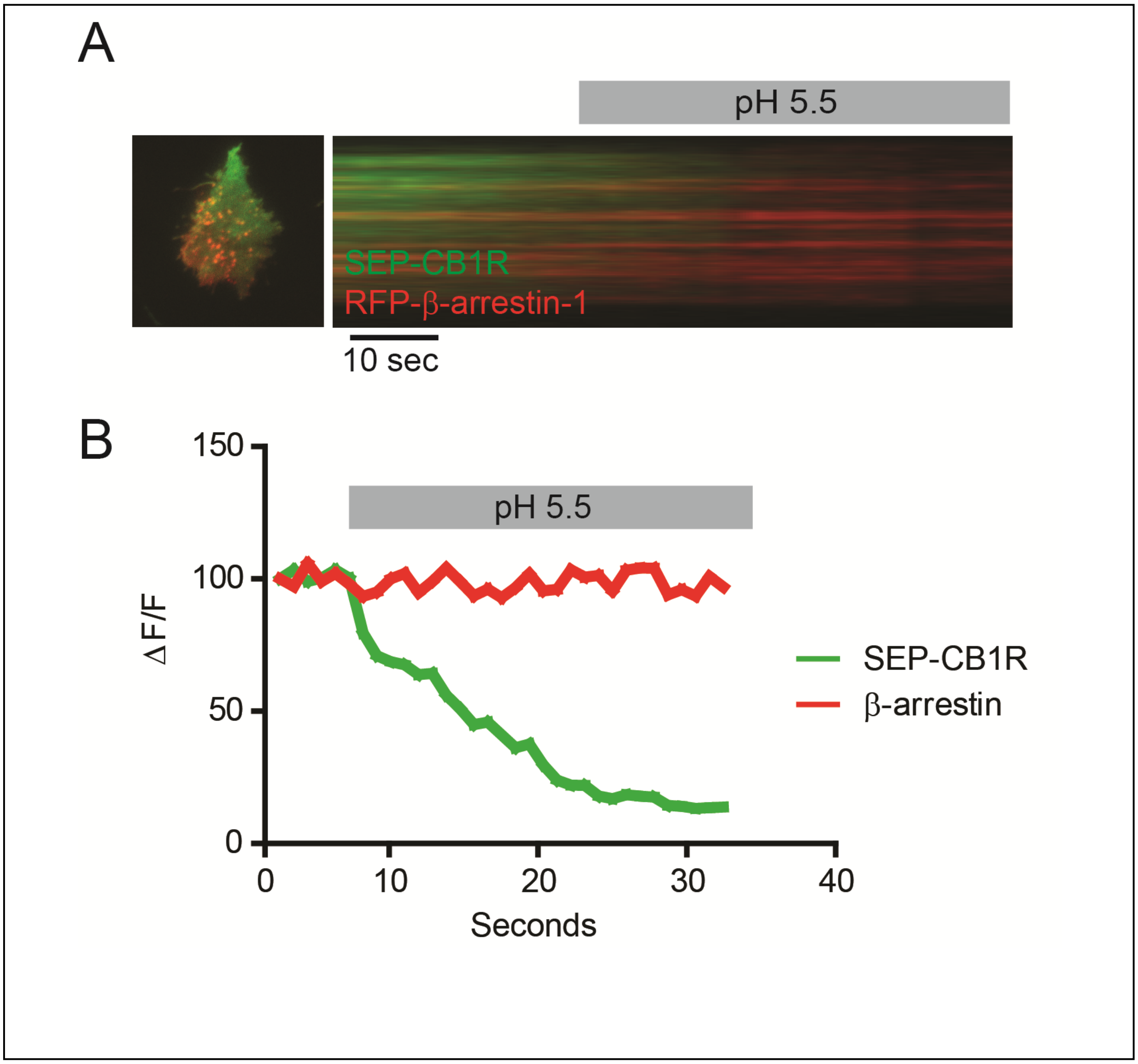

Supplementary Figure 1. CB1R clusters dwell at the plasma membrane. HEK293 cells co-expressing SEP-CB1Rs and RFP-β-arrestin-1 were incubated with 5 μM WIN 55,212-2 and imaged under TIRF microscopy. (A) Single frame shows SEP-CB1Rs clusters colocalized with RFP-β-arrestin-1 after 10 minutes incubation. Kymograph shows changes in SEP-CB1R fluorescence intensity from endocytic clusters while pH in the imaging media is reduced by addition of MES pH5.5. (B) Changes in fluorescence intensities from cell in A indicating rapid quenching of SEP-CB1R intensity and no change in RFP-β-arrestin-1.

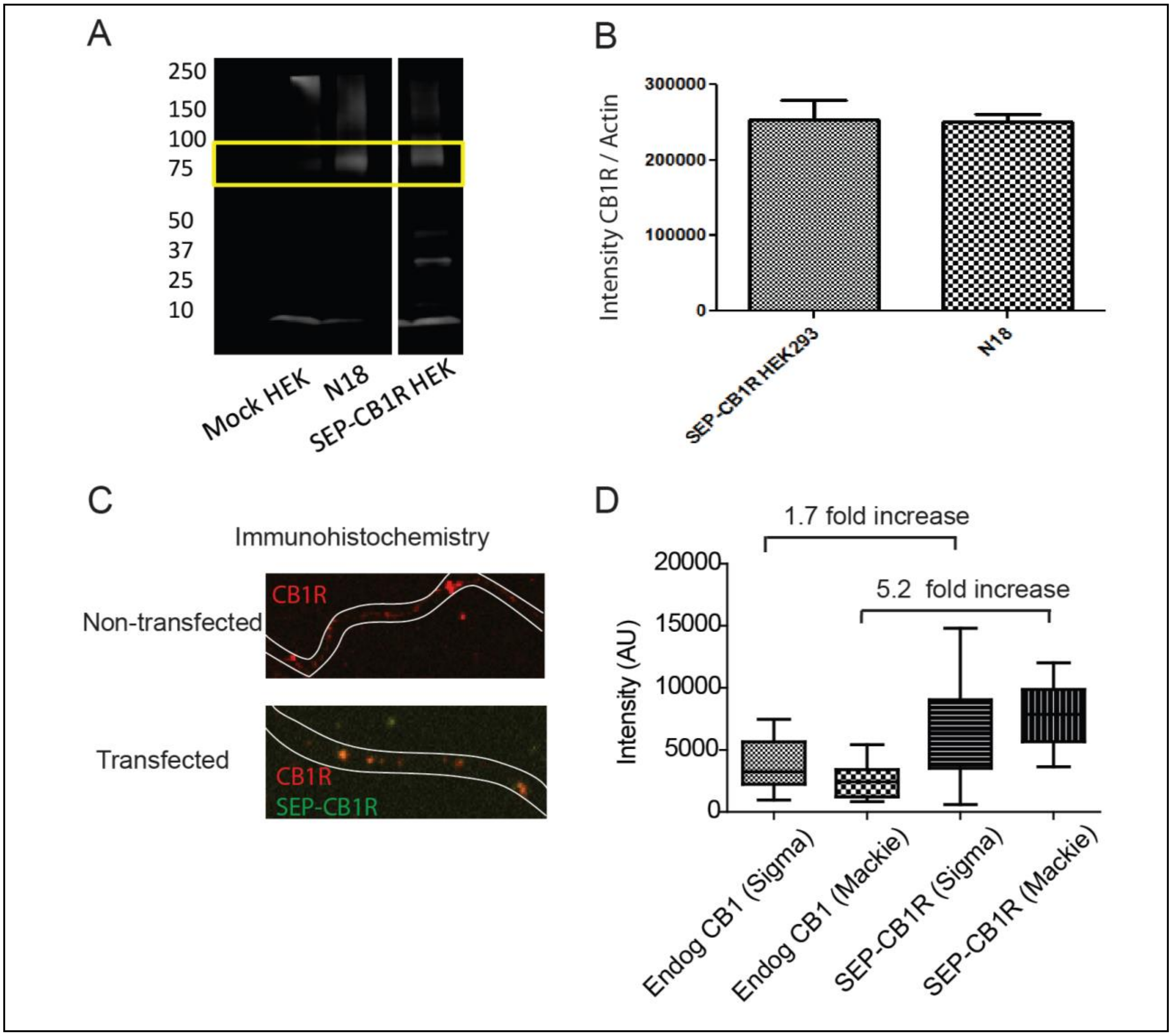

Supplementary Figure 2. SEP-CB1R is localized and expressed at comparable levels to the endogenous receptors. (A) Expression levels of CB1Rs were compared by immunoblot from untransfected HEK293 cells, N18 neuroblastoma cells expressing endogenous receptors and HEK293 cells stably expressing SEP-CB1Rs. Equal protein was loaded. (B) Analysis of CB1R intensities were normalized to actin (n=3). Error bars represent SEM. (C) Immunohistochemistry from dissociated hippocampal neurons show endogenous CB1R (top) and over-expressed levels (bottom). (D) Average fluorescence intensities from multiple dendrites were measured and compared for two different antibodies (Sigma and Mackie(L)). Immunofluorescence of SEP-CB1R over-expression is ~3 to ~7 times higher than endogenous CB1R. Fluorescence intensity from individual CB1R puncta was quantified and compared between samples and antibodies (n=4-7 experiments, 5-10 cells/experiment).

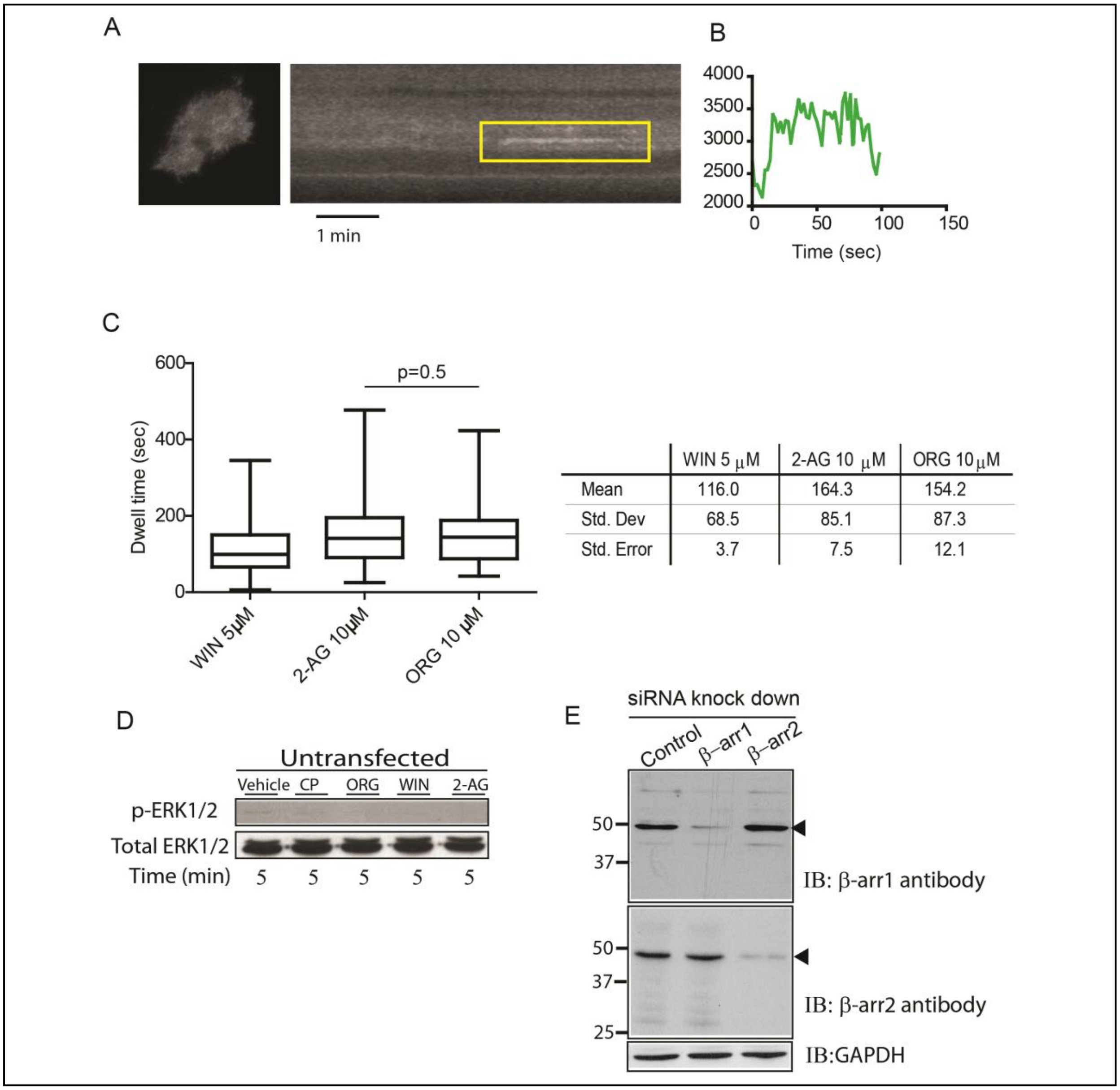

Supplementary Figure 3. ORG27569, an ago-allosteric modulator, elicits long CB1R dwell times and ERK phosphorylation in the absence of CB1Rs. (A-B) HEK293 cells stably expressing SEP-CB1Rs were first washed in imaging media and then incubated with 10 μM ORG27569 and imaged under TIRF microscopy. ORG27569 elicited a small number of Individual endocytic events with similar kinetics to 2-AG. (C) Box and whiskers plot showing dwell times of a single endocytic event elicited by 5 μM WIN 55,212-2, 10 μM 2-AG and 10 μM ORG27569 (n=336 events/12 cells, 244 events/9 cells and 52 events/respectively). (D) Untransfected HEK293 exposed to CP55940, ORG27569, WIN 55,212-2, and 2-AG showed no effect on ERK1/2 phosphorylation. (E) The representative western blot depicts isoform-specific knockdown of endogenous β-arrestin 1 and 2 expression by siRNAs. Immunodetectable levels of GAPDH are shown as loading controls.

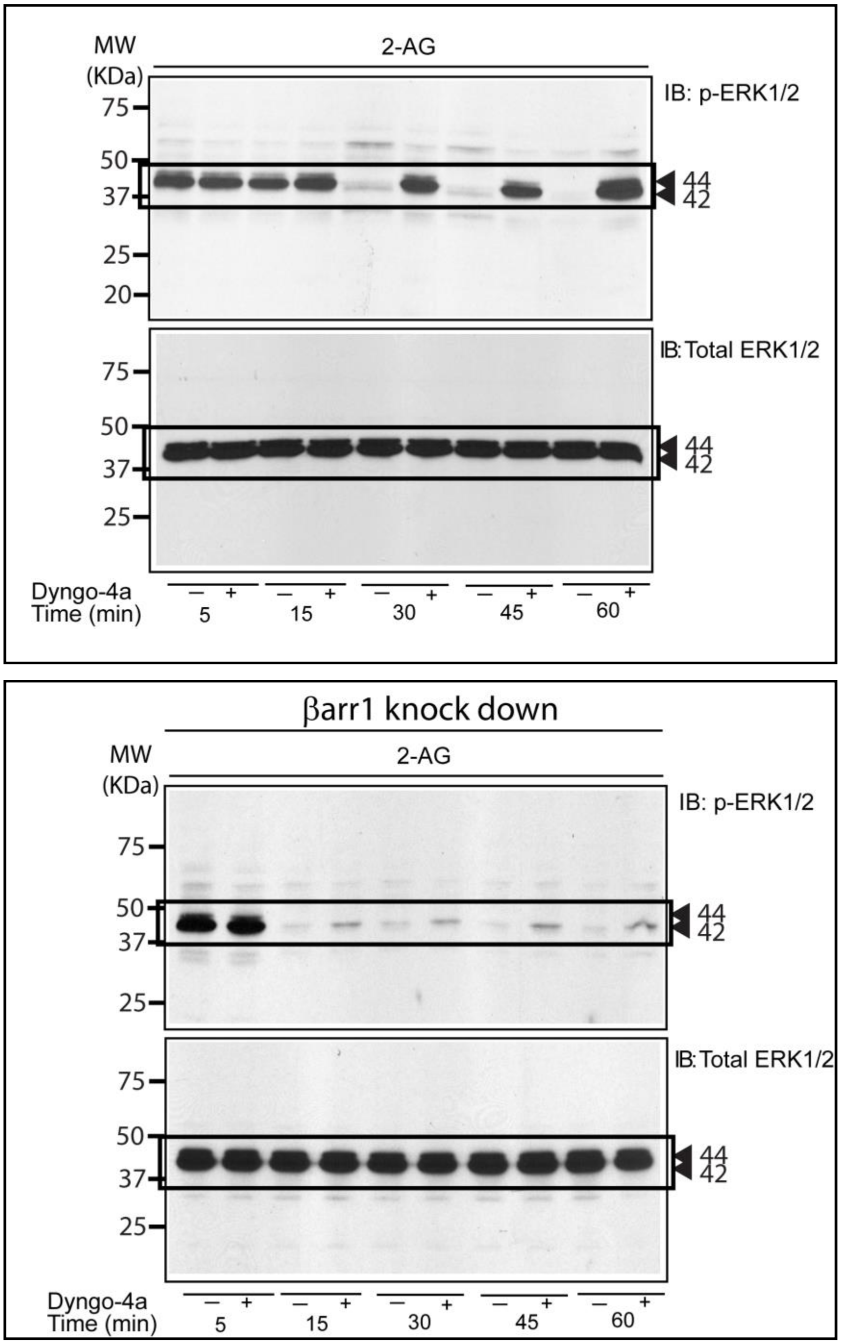

Supplementary Figure 4. The original scanned key western blots. The Figures 5B and 5E were extrapolated from these blots.
